# Assessing the accuracy of self-reported self-talk

**DOI:** 10.3389/fpsyg.2015.00570

**Published:** 2015-05-06

**Authors:** Thomas M. Brinthaupt, Scott A. Benson, Minsoo Kang, Zaver D. Moore

**Affiliations:** ^1^Psychology, Middle Tennessee State UniversityMurfreesboro, TN, USA; ^2^Health and Human Performance, Middle Tennessee State UniversityMurfreesboro, TN, USA

**Keywords:** Self-Talk Scale, self-reports, inner experience, personality assessment, individual differences

## Abstract

As with most kinds of inner experience, it is difficult to assess actual self-talk frequency beyond self-reports, given the often hidden and subjective nature of the phenomenon. The Self-Talk Scale (STS; Brinthaupt et al., 2009) is a self-report measure of self-talk frequency that has been shown to possess acceptable reliability and validity. However, no research using the STS has examined the accuracy of respondents’ self-reports. In the present paper, we report a series of studies directly examining the measurement of self-talk frequency and functions using the STS. The studies examine ways to validate self-reported self-talk by (1) comparing STS responses from 6 weeks earlier to recent experiences that might precipitate self-talk, (2) using experience sampling methods to determine whether STS scores are related to recent reports of self-talk over a period of a week, and (3) comparing self-reported STS scores to those provided by a significant other who rated the target on the STS. Results showed that (1) overall self-talk scores, particularly self-critical and self-reinforcing self-talk, were significantly related to reports of context-specific self-talk; (2) high STS scorers reported talking to themselves significantly more often during recent events compared to low STS scorers, and, contrary to expectations, (3) friends reported less agreement than strangers in their self-other self-talk ratings. Implications of the results for the validity of the STS and for measuring self-talk are presented.

## Introduction

Conducting research on the psychology of inner experiences is an interesting and challenging activity. Because the phenomena of interest may be covert, hidden, or completely unobservable by an outside agent, researchers must rely primarily on the introspection and self-reports of participants. Several of the other papers in this special issue address ways to overcome some of the limitations of and provide complements to self-report with respect to different kinds of inner experiences. In the present paper, we describe three studies designed to assess the accuracy of self-reported self-talk.

There is a long history of research interest in the veridicality of self-reports (e.g., Schoeneman, 1981; Shrauger and Osberg, 1981; Moskowitz, 1986; Brinthaupt and Erwin, 1992; Vazire and Wilson, 2012). Interest in the accuracy of self-reports covers a broad range of phenomena, particularly behaviors that might be expected to show socially desirable responding effects. For example, Gatersleben et al. (2002) found that self-reports of pro-environmental behaviors were only weakly related to actual household energy use. In a meta-analysis of the validity of self-reported drug use, Magura and Kang (1996) found that underreporting was a prevalent issue. People have also been found to underestimate their frequency of sedentary behaviors (e.g., Klesges et al., 1990) and underestimate their dietary intake (e.g., Palaniappan et al., 2003).

With respect to reporting about specific self-related phenomena, the literature finds that self-reports of personality traits generally show moderate agreement with observations from others and behavioral indicators (e.g., Mehl et al., 2006; Vazire and Mehl, 2008; Back et al., 2009). Research also suggests that people are more accurate when reporting about personality disorders than when reporting about mental disorders, possibly because the former are seen as part of one’s self-definition and as not as something that is unacceptable or a reflection of a disturbance (e.g., Oltmanns and Turkheimer, 2006). Furthermore, in domains that reflect more positive personal behaviors or characteristics, research suggests that when people are uncertain, they are likely to report or demonstrate having personality traits that are seen by others as positive, such as prosocial characteristics (Chin et al., 2012).

Research on maladaptive cognitive and affective variables supports the idea that self-reports are generally similar to the reports of knowledgeable informants (e.g., South et al., 2011), although some clinical issues, such as narcissistic personality disorder, appear to be particularly susceptible to self-other disagreement (Klonsky et al., 2002). Carlson et al. (2013) found that self-reports showed greater validity than informant reports for internalizing personality disorders (such as showing neuroticism, anxiety, or obsessive–compulsive tendencies). Alternatively, they found that informant reports showed greater validity than self-reports for personality disorders that are more externalizing in nature (e.g., being disagreeable, aggressive, or narcissistic).

Whereas self-talk appears to be primarily an internalizing rather than externalizing phenomenon, it is unclear how people perceive the appropriateness of their self-talk. Perceptions of self-talk appropriateness are likely to differ depending on whether we consider the frequency of self-talk (e.g., how often people talk to themselves) or its affective (i.e., positive and negative) content. Researchers find that people are frequently biased toward self-enhancing self-perceptions, especially individuals who show high levels of narcissism (Schriber and Robins, 2012). However, it is unclear the extent to which self-talk is seen as a socially desirable or undesirable characteristic. Brinthaupt et al. (2009) found that self-reported self-talk was only weakly related to a measure of social desirability. That finding suggests that self-talk frequency may not be seen in a particularly negative way among respondents.

Maladaptive or dysfunctional self-talk content – unrealistic, irrational, or excessively negative – has been a focus of cognitive-behavioral therapists for many years (e.g., Beck, 1976; Glass and Arnkoff, 1994). An implication of this focus is that certain kinds of self-talk may be seen by people as less socially desirable. If this is the case, then the accuracy of certain kinds of self-reported self-talk, such as self-critical self-statements, may be negatively associated with perceptions of inappropriateness or social undesirability. More positive or affectively neutral self-talk, such as self-managing self-statements, should be less strongly related to those perceptions. People’s reports of the frequency of different kinds of self-talk might therefore be affected by their beliefs or presuppositions about how maladaptive or dysfunctional it is (Hurlburt and Heavey, 2015).

Self-talk frequency is related to a wide variety of self-regulatory behaviors (e.g., Mischel et al., 1996; Carver and Scheier, 1998; Leary, 2004). With the increasing interest in self-talk as a psychological phenomenon across multiple domains (Beck, 1976; Kendall et al., 1989; Hardy et al., 2009; Winsler et al., 2009; Hurlburt et al., 2013), it is crucial that data be collected that examine the accuracy of self-reported self-talk. Among the important questions here include (1) the extent that people’s reports of their self-talk frequency correspond to actual behavioral instances of self-talk across a variety of everyday situations or circumstances and (2) whether people’s awareness of their self-talk reflects their self-reported frequency of self-talk instances, as assessed at different times, or through different kinds of data collection.

## Self-Talk and the Self-Talk Scale

The development and initial validation of the Self-Talk Scale (STS; Brinthaupt et al., 2009) permits researchers to examine individual differences in self-talk frequency. The STS is general measure of self-talk that is applicable to a broad range of self-regulatory behaviors and situations. The scale consists of 16 items rated on a five-point frequency scale (1 = *never*, 5 = *very often*) using the common stem “I talk to myself when…” Brinthaupt et al. (2009) showed that the STS has a structure consisting of one higher-order factor (overall self-talk) and four primary factors (self-critical, self-reinforcing, self-managing, and social-assessing self-talk), along with acceptable test–retest stability and internal consistency.

Self-critical self-talk is generally associated with negative events (e.g., “I feel ashamed of something I’ve done” or “Something bad has happened to me”). Self-reinforcing self-talk focuses on positive events (e.g., “I’m really happy for myself” or “I’m proud of something I’ve done”). Self-managing self-talk pertains to general self-regulation (e.g., “I’m mentally exploring a possible course of action” or “I’m giving myself instructions or directions about what I should do or say”). Finally, social-assessing self-talk refers to people’s social interactions (e.g., “I want to replay something that I’ve said to another person” or “I want to analyze something that someone recently said to me”).

In their STS validation work, Brinthaupt et al. (2009) found negative relationships between social-assessing and self-critical self-talk and self-esteem, as well as a positive association between self-reinforcing self-talk and self-esteem. Frequent self-talkers (i.e., those scoring in the upper quartile on the total STS) also scored higher than infrequent self-talkers (lowest quartile) on need for cognition and obsessive–compulsive tendencies.

Additional research using the STS shows more frequent self-talk among adults who reported having had an imaginary companion in childhood or who grew up as an only child compared to those who did have those experiences (Brinthaupt and Dove, 2012) and a high negative correlation between loneliness and mental health in more frequent self-talkers (Reichl et al., 2013). Research using an adaptation of the STS (Shi et al., 2015) has found that individuals with high public speaking anxiety were cognitively “busier” (i.e., reported higher levels of several kinds of self-talk) than those with low anxiety as they prepared for an upcoming speech. Finally, research has supported the use of the STS response format and the use of the STS total score as a unidimensional measure of self-talk frequency (Brinthaupt and Kang, 2014), as well as shown a similar factor structure in a cross-cultural comparison (Khodayarifard et al., 2014).

In summary, the STS possesses good psychometric properties, and scores on the measure have been associated with a wide range of interesting phenomena. Clearly, the scale shows promise as a measure of self-reported self-talk. However, an important question is to what extent the self-talk that STS respondents report reflects their actual self-talk frequency. As Hurlburt and Heavey (2015) have shown, respondents’ reports of any kind of inner experience can be problematic, particularly when those reports are retrospective. Previous reviews of self-talk measures (e.g., Glass and Arnkoff, 1994; Uttl et al., 2011) propose that validity can be examined through the use of multiple measures and assessment occasions. There are several interesting theoretical and research implications related to the analysis of the accuracy of self-reported self-talk and to the different self-talk functions. In this paper, we report the results of three studies examining the correspondence between self-reports of self-talk using the STS and behavioral or observer indicators of self-talk. In addition, we examine the four STS sub-scales and how these are associated with different levels of accuracy or agreement.

## Study 1: STS Scores and Recent Self-Talk Experiences

As with other domains of personality self-knowledge (Back and Vazire, 2012), the accuracy of self-reports of one’s self-talk frequency is likely to be affected by explicit and implicit information processing, the salience of relevant behavioral instances, accessibility to ongoing inner experiences, and information from other people (Hurlburt et al., 2013). It is likely that people rely on a wide variety of information sources and presuppositions when completing the STS. We would expect that respondents who notice or recall more situations in which they have talked to themselves in the past should be likely to report more frequent self-talk. Assuming that people respond to the STS based on an aggregation of behavioral instances, cognitive heuristics, presuppositions, times, and situations associated with self-talk (Kenrick and Funder, 1988; Hurlburt et al., 2013), self-talk likelihood in sample situational instances should be positively associated with self-reports of typical self-talk.

In Study 1, participants first completed the STS and then, at a later time, completed a revised version of the measure. We expected that, if the STS assesses respondents’ awareness of or assumptions about their typical self-talk frequency, then high STS scorers would report being more likely than low STS scorers to talk to themselves when specific STS-related situations occur. Thus, we predicted that previous overall and subscale STS scores, based on how often people report typically talking to themselves, would be positively and significantly correlated with their reports of the frequency of self-talk in response to relevant situations that had recently occurred.

### Method

#### Participants

Through the cooperation of three faculty members from human sciences, speech, and English departments, 83 students (27 men, 56 women) from a large southeastern U. S. public university completed the materials. Students’ ages ranged between 18 and 31 years (*M* = 20.01, SD = 2.23). For two of the instructors, students completed the surveys during their normal class time, with the other instructor permitting students to complete the measures outside of class and returning them at the next class period. Students volunteered to participate, completed an informed consent form, and received a small number of extra credit points for their participation at the discretion of their instructors. This study (as well as Studies 2 and 3 reported later) received IRB approval prior to data collection.

#### Materials

Students completed the original STS early in the academic term and then, approximately 6 weeks later, completed a revised version of the measure. STS internal consistency coefficients were acceptable for the overall scale (*r* = 0.92) and the subscale (*r*s ranging from 0.79 to 0.87). For the modified, “recent experience” version of the STS (reSTS), participants indicated whether they had recently experienced any of the 16 situations from the original STS, as well as whether they had engaged in self-talk during those experiences. The reSTS wording of the items was modified from the original STS to reflect the past-tense nature of the question posed. For example, we changed the original STS item “I want to replay something I’ve said to another person” to “I wanted to replay something I said to another person” for the reSTS. Wording was only changed as much as necessary to make the items grammatically correct in the past tense. The order of the items in the reSTS was the same as the original STS.

Participants received the following instructions for completing the reSTS: “check all that apply; over the past 2 days, I have been in a situation where…” They rated the 16 items corresponding to the STS items (e.g., “Something good happened to me” and “I was really upset with myself”) in terms of whether that situation had occurred (yes/no). For each situation that had occurred, students then indicated (yes/no) whether they had talked to themselves “(either silently or aloud) about that situation as it occurred or shortly after it occurred.” From these data, we calculated the total number of STS-related situations that had occurred over the past 2 days (possible range 0–16), the number of situations that had occurred for each of the four STS subscales (possible range 0–4), and the total and subscale ratios of situations where students reported talking to themselves when those situations had occurred (possible range 0.000–1.000). For the latter subscale ratios, when no instances of subscale situations occurred, values were set to missing.

#### Procedure

As noted earlier, participants received materials twice in a semester during regular class time. At the first session, they received a consent form, a paper copy of the STS, and a demographic form that included questions about age and gender. Students also included their student ID numbers on the form, in order to permit matching of data from the second session. At the second data collection period, we visited the same classrooms, and the participating students completed the reSTS on paper. After the data from the second session were collected, we thanked and debriefed the participants. The surveys, including the consent form, took no more than 10 min to complete.

### Results and Discussion

**Table 1** provides the descriptive statistics for the reSTS measures. These data indicate that, in the 2-day period used for assessment, approximately 67% of the 16 STS situations had occurred. Of these situations, self-managing ones were most frequent, whereas self-critical were least frequent. The data also indicated that participants reported self-talk associated with over 80% of STS situations that had occurred. The self-talk ratio was highest for the self-managing and lowest for the self-reinforcing situations. These data support the idea that there are numerous daily opportunities for people to talk to themselves and that they frequently report doing so when they encounter those situations.

**Table 1:** Study 1: Descriptive statistics for reSelf-Talk Scale (STS) measures.

Item	Mean	SD	*n*
reSTS event frequency			
Overall	10.78	3.66	83
Self-critical	2.04	1.44	83
Self-reinforcing	2.70	1.36	83
Self-managing	3.10	1.10	83
Social-assessing	2.95	1.20	83
reSTS self-talk ratio			
Overall	0.815	0.249	83
Self-critical	0.782	0.363	71
Self-reinforcing	0.695	0.394	74
Self-managing	0.870	0.272	83
Social-assessing	0.810	0.334	80

reSTS refers to revised STS items; event frequency refers to the number of STS-related situations (out of 16 for overall, out of four for each subscale) that had occurred; self-talk ratio refers to the proportion of instances of STS situations that had occurred in which participants reported that associated self-talk had also occurred.

**Table 2** provides the correlations among the major measures for both testing sessions. As the table indicates, STS total scores were positively correlated with the reSTS overall ratio. In other words, frequent self-talkers reported talking to themselves more often than infrequent self-talkers when STS-related situations recently occurred. Each of the STS subscales were also positively and significantly related to their corresponding reSTS ratio scores, with the self-critical and self-reinforcing scores showing stronger relationships than the self-managing and social-assessing scores. In addition, STS total scores were positively related to the total number of STS situations that had occurred in the previous 2 days, *r*(81) = 0.395, *p* < 0.001.

**Table 2:** Study 1: Correlations among the STS and reSTS measures.

STS:	Total	Self-critical	Self-reinforcing	Self-managing	Social-assessing
reSTS ratio					
Overall	0.446^∗∗∗^	0.331^∗∗^	0.356^∗∗∗^	0.460^∗∗∗^	0.372^∗∗∗^
Self-critical	0.377^∗∗∗^	0.373^∗∗∗^	0.231	0.332^∗∗^	0.372^∗∗∗^
Self-reinforcing	0.412^∗∗∗^	0.202	0.439^∗∗∗^	0.417^∗∗∗^	0.340^∗∗^
Self-managing	0.231^∗^	0.134	0.170	0.257^∗^	0.225^∗^
Social-assessing	0.323^∗∗^	0.221^∗^	0.218	0.371^∗∗∗^	0.293^∗∗^

^∗^*p* < 0.05, ^∗∗^*p* < 0.01, ^∗∗∗^*p* < 0.001; reSTS ratio refers to the proportion of instances of STS situations that had occurred in which participants reported that associated self-talk had also occurred.

These data provide good support for the expectation that typical self-reported self-talk scores, assessed 6 weeks earlier, would correspond to self-reports of recent examples of instances where self-talk could occur. There are at least two possible explanations for these results. First, general assessments of people’s typical self-talk frequency are an accurate representation of the frequency with which they actually talk to themselves across a variety of specific and recent self-regulatory situations. For example, people who report that they frequently talk to themselves when something bad or good happens to them also report having done so when something bad or good has recently happened. Second, it is possible that people customarily recall salient examples of self-talk (e.g., within the previous few days) when they assess the frequency of their typical self-talk using the STS. If this recall pattern occurs, then we have simply assessed the situations that respondents are already relying on when they rate their typical self-talk patterns using the original STS.

The fact that STS scores were significantly associated with the occurrence of STS-related situations suggests that frequent self-talkers are more aware of or responsive to a variety of self-regulatory situations than infrequent self-talkers. Perhaps self-talk frequency is positively associated with a greater overall sensitivity to one’s intrapersonal and interpersonal experiences. Of course, we cannot determine the extent to which participants were recalling their previous STS responses when rating their self-talk in recent situations. If they were able to recall their earlier STS responses and were motivated to be consistent, then this could account for the observed relationships. Future research could address these possible explanations.

In summary, Study 1 provided data to suggest that (1) the self-talk situations included in the STS are frequently reported occurrences in people’s lives, (2) self-talk is reported to frequently occur in those situations, and (3) respondents’ self-reports of their typical self-talk generally agree with their reports about specific related experiences. Although these results were encouraging, a variety of alternative explanations could not be addressed or controlled in this study.

## Study 2: Experience Sampling Study of Current Ongoing Self-Talk

A limitation of Study 1 is that participants’ recall of relevant instances and their accompanying self-talk was based on events from the past 48 h. Because of the time lag involved, the extent of agreement between original STS scores and recent instances of self-reported self-talk may have been constrained. A stronger test of the self-report accuracy question would involve very recent experiences that should be more salient and accessible to the participants.

The experience sampling method (ESM) has proven to be a reliable and valid method for measuring a wide range of inner experiences (Csikszentmihalyi and Larson, 2014). In Study 2, we utilized ESM methods to compare general STS reports with the self-talk that is reported to occur in response to current experiences. In particular, we examined the self-talk patterns of those who fell in the upper and lower quartiles of the STS total scores. These self-reported *frequent* and *infrequent* self-talkers were prompted periodically on their smart phones for 5 days to indicate whether any of the 16 STS situations had occurred within the past 2 h. If so, they then reported whether or not they had talked to themselves about that instance.

We expected that, when instances of STS-related situations occurred, previously identified frequent self-talkers would report more accompanying self-talk than would the infrequent self-talkers. In other words, across the array of situations included in the STS, more overall and subscale self-talk should be reported during those situations for the frequent than the infrequent self-talkers.

### Method

#### Participants

Using data collected approximately 1 month earlier from the university’s General Psychology pretesting research pool, we recruited 35 participants (8 male, 27 female) from the upper (*n* = 20) and lower (*n* = 15) quartiles of total STS scores. The lower quartile participants (*M* = 19.93, SD = 6.46) differed significantly on STS scores from the upper quartile participants (*M* = 50.70, SD = 5.39), *t*(33) = 15.34, *p* < 0.001. Participants ranged in age from 18 to26 years (*M* = 18.66, SD = 1.78). With respect to ethnicity, 74% of the participants were Caucasian and 11% were African–American. In order to participate in the study, students were required to possess an operable smart phone that could receive text messages and connect to the Internet.

#### Materials and Procedure

Using a modified version of the STS, we asked participants about their very recent or currently ongoing activities and whether those activities were associated with a self-reported instance of self-talk. Due to software limitations, the order of the questions was the same as the original STS. We modified the STS items in two key ways for this study. First, each item included the phrase “Over the last *two hours*, I have been in a situation where…” Care was taken to change only what was necessary to match tense for the complete phrase. Second, participants simply answered *yes* or *no* to whether each of the 16 situations had recently occurred. For items answered *yes*, a follow-up yes/no question appeared: “Did you talk to yourself (either silently or aloud) during or immediately after the situation occurred?”

We used a commercial survey hosting web-service to administer materials and organize response data. A free Gmail account and a paid account to Right Inbox scheduled the text messages. Right Inbox is a web-browser extension that allows e-mail drafts in the Gmail web-based e-mail client to automatically send themselves at scheduled times. Over a 5-day period, participants received 25 text message prompts (five each day) during a 10-h daily period (10 am–8 pm). Participants used the same assigned number to identify themselves at the start of each of the surveys.

There were 32 possible yes/no questions on each survey text prompt. Possible scores on these measures ranged between 0–16 for the first questions (situations that had recently occurred) and the follow-up (talking to oneself if the situation had occurred). Thus, over the course of the study, there were 400 possible instances where an STS situation could have occurred and where participants could have reported talking to themselves. As in Study 1, we calculated the total and subscale ratios of situations where students reported talking to themselves when those situations had occurred (possible range 0.000–1.000). For the latter subscale ratios, when no instances of situations occurred, values were set to missing.

A random number generator provided scheduled times for contacting research participants. Twenty-five numbers between 0 and 119 were selected at random, and the corresponding number of minutes was added to the start time for each of the five 2-h blocks. For example, a 63 selected for a 10 am–12 pm block would add 63 min to the 10 am start time, so that the text message would be scheduled to be sent at 11:03 am. In the selection of these contact times, we ensured that no two consecutive prompt times occurred within 30 min of each other.

E-mails were converted to text messages using standardized e-mail addresses issued by cell phone carriers to each phone number. Standard format for an assigned “e-mail to SMS” e-mail address is the 10 digits of the phone number “at” a domain hosted by the carrier. For example, a Verizon phone number, (931) 555-1234, can receive as text messages any e-mail sent to 9315551234@vtext.com. We recorded the appropriate text message addresses for all research participants and sent a pilot message before the study began in order to identify and correct any address issues.

Each unique link that led to the survey directed participants to a new “collector,” or a new identifiable instance of the survey. This allowed data from each of the 25 surveys to remain separate from one another, while the participant’s ID number allowed us to collate these data. Additionally, time signatures on each collector allowed for sorting the data by time-order.

At an orientation session at the start of the study, we briefed participants on the details of the ESM, including the scheduling of the text messages and that surveys would need to be completed online and within 2 h of receiving the link to each survey. Each participant provided their phone number and carrier for receiving text messages. After initial testing of the text message system and phone compatibility, students received links to surveys through text messages, beginning the following Monday. Participants agreed to receive five text messages a day for five consecutive days. All students participated during the same 5-day period and received all text messages at the same intervals.

The links in the text messages directed participants to a survey hosted on a commercial survey website. Surveys remained open for 2 h after receiving the text message link. This gave participants some flexibility in answering the survey without allowing excessive overlap of reporting periods. After 2 h, the link instead directed participants to a page explaining that the survey was closed.

Over the 2 weeks following the testing week, participants returned for debriefing and to receive credit for participating in the study. The exit survey included demographic items (ID number, age, gender, and ethnicity) and a yes/no question about whether they had ever considered their level of self-talk prior to the study. In addition, participants rated seven items regarding their experiences during the ESM study, using a five-point scale (1 = *very little*, 5 = *very much*). These items included the difficulty in determining and recalling whether self-talk had occurred when prompted, the difficulty of completing the survey on time, whether the study questions and directions were clear, whether the survey responses captured most daily instances of self-talk, and whether their awareness of self-talk increased after participating in the study.

### Results and Discussion

Results showed that participants took an average of 20.32 min (SD = 24.74) to respond to the receipt of the text messages. Frequent and infrequent self-talkers did not differ significantly on this measure [*t*(34) = 0.186, *p* = 0.85]. Participants reported that approximately 23% of the 400 possible STS-related situations had occurred (*M* = 91.46, SD = 47.02) over the 5-day period. In addition, participants reported talking to themselves 65% of the time when the overall STS-related situations had occurred. With respect to the STS subscales, participants reported talking to themselves 72% of the time for the self-critical and self-managing situations, 63% for the social-assessing situations, and 51% for the self-reinforcing events.

Data for the major measures by frequent and infrequent self-talkers are presented in **Table 3**. As the table shows, the groups did not differ significantly on the number of STS-related situations that had occurred over the 5 days or the number of those situations in which self-talk was reported to have occurred. These results suggest that the everyday experiences related to the topics included in the STS are similar for frequent and infrequent self-talkers. However, as expected, frequent self-talkers reported a significantly higher overall proportion of talking to themselves when STS situations had occurred than did infrequent self-talkers. Examination of the STS subscales showed that frequent self-talkers differed significantly from infrequent self-talkers in their reports of self-reinforcing and self-managing self-talk. Infrequent self-talkers reported being least likely to talk to themselves during self-reinforcing situations, whereas frequent self-talkers reported being most likely to talk to themselves in response to self-managing situations.

**Table 3:** Study 2: Comparison of infrequent and frequent self-talkers on major measures.

	Infrequent (*n* = 15)		Frequent (*n* = 20)			
	Mean	SD	Mean	SD	*t*	*p*
# of STS situations	81.73	54.92	98.75	40.05	1.061	0.296
# of situations w/self-talk	53.60	54.37	76.85	43.14	1.411	0.167
Self-Talk Ratio						
Overall	0.538	0.253	0.734	0.216	2.465	0.019
Self-critical	0.632	0.269	0.779	0.236	1.722	0.094
Self-reinforcing	0.355	0.320	0.619	0.221	2.889	0.007
Self-managing	0.598	0.317	0.808	0.193	2.431	0.021
Social-assessing	0.586	0.371	0.655	0.339	0.558	0.581

Self-talk ratio refers to the proportion of instances of STS situations that had occurred in which participants reported that associated self-talk had also occurred. Reported ratio values reflect the average of the proportions for the individual members of each group.

The final analyses examined the post-study survey data and how participating in the study affected people’s attention to their self-talk. Twenty-seven of the 35 participants (13 infrequent, 14 frequent self-talkers) completed this survey. With respect to having ever considered their level of self-talk prior to the study, 67% of the infrequent self-talkers reported no and 62% of the frequent self-talkers reported yes [*X*^2^(1) = 1.99, *p* = 0.158). The two groups did not differ significantly on any of the survey measures. Thus, the participants’ reports of their study experiences were similar regardless of their self-talk frequency status.

In summary, there was little evidence that the frequent and infrequent self-talkers differed in how often STS-related situations occurred during the 5 days of the study or that the two groups experienced the study methodology differently. However, frequent self-talkers reported being significantly more likely to talk to themselves when those situations occurred than did the infrequent self-talkers. This result provides additional support for the validity of the STS and of individuals’ self-reports of their typical self-talk frequency.

It is interesting that the infrequent self-talkers (as identified by their scores on the STS from the previous month) reported talking to themselves when STS situations occurred nearly 54% of the time. This is higher than the percentage of self-talk frequency based on this group’s mean STS scores (19.93/64 or 31%). The frequent self-talkers reported talking to themselves when STS situations occurred 73% of the time, which is very similar to their mean STS score percentage (50.70/64 or 79%). It appears that those who rate themselves as infrequent self-talkers still report talking to themselves around half of the times shortly after STS-related situations occur, but that they under-report that frequency when completing the STS. It is also possible that there was a regression to the mean effect for the infrequent and frequent self-talkers. Because we selected lower- and upper-quartile STS scorers for the groups, their later situation-specific self-talk reports might be more likely to increase (decrease).

Our methodology in Studies 1 and 2 did not permit an assessment of the amount of time spent talking to oneself when STS situations occurred. We only asked participants to indicate whether they had talked to themselves in response to the situation occurring. Future research could examine the length, depth, or salience of one’s self-talk following the occurrence of these situations. Frequent self-talkers would be expected to show large differences on these self-talk characteristics compared to infrequent self-talkers. It is possible that degree of cognitive processing of events contributes more to people’s general assessments of their self-talk frequency (and whether they are categorized as frequent or infrequent self-talkers) than the occurrence of situations that prompt self-talk.

The first two studies provided good support for the prediction that self-reports of typical self-talk frequency using the STS are accurate. Analysis of the STS subscales revealed some interesting qualifications to the general trend. Study 1 showed that all the STS subscales were significantly correlated with recent situations (which had occurred within the past 2 days). However, Study 2 showed that frequent and infrequent self-talkers did not differ in their self-reported self-critical and social-assessing self-talk frequency in specific situations that had occurred very recently. It is possible that these categories of self-talk are more difficult to estimate accurately. It is noteworthy that both of these subscales assess more negative than positive self-talk instances (Brinthaupt et al., 2009). Individuals appear to be most accurate in judging their typical self-reinforcing self-talk frequency. Once again, research examining the length, depth, or salience of one’s self-talk would provide important information about the memorability of different kinds of self-talk and how that memorability contributes to assessments of one’s typical self-talk frequency.

## Study 3: Self and Other Ratings of Self-Talk Frequency

Another way to examine the validity of self-reported self-talk is to compare self-reports to the reports of knowledgeable others. Unfortunately, there are several reasons why self-talk might not be easy to monitor in another person. This task may be similar to comparing self- and other-reports of a person’s internal physiological states (e.g., the severity of a headache and one’s hunger status). In these cases, there is very little information that an observer could rely on to provide an accurate assessment. In addition, due to self-presentation or impression management reasons (such as concerns about one’s “sanity”), both silent and aloud self-talk are probably more likely to be used when a person is alone than with others. By its very nature, self-talk is self-directed speech that appears to not be intended for the ears of other people. Due to issues of attention or focus, it may also be more difficult mechanically and socially to engage consistently in self-talk in the presence of another person than when alone. Thus, the ability for an observer to assess accurately the self-talk frequency of a target person may be fundamentally limited.

Despite the intrapersonal nature of self-talk, there is evidence that there are interpersonal aspects of the phenomenon. For example, research shows that children (and to a lesser extent adults) will engage in more private speech when performing tasks in the presence of others than when alone (McGonigle-Chalmers et al., 2014). There may be times when people talk to themselves (either silently or aloud) in the presence of others for strategic reasons (e.g., to convey an emotional response or to indicate that one is actively thinking about an issue). Thus, there appear to be some interpersonal aspects of the inner experience of self-talk. If this is true, then other people, particularly those with extensive knowledge of and experience with the respondent, should show high levels of agreement with that respondent’s self-reported self-talk frequency.

There is research support for the idea that other people can contribute to the accuracy of one’s self-views. For example, Srivastava (2012) noted that personal attributes that are reputational in nature (such as social status or likeability) are strongly affected by how one is seen by others. Because covert or silent self-talk is a highly internalized phenomenon, these results suggest that self-reports of self-talk might be more valid than the reports of informants. On the other hand, people who engage in frequent private speech (out loud self-talk) may cue informants about their likely inner speech (silent self-talk) frequency.

In the realm of personality pathology, South et al. (2011) found that, with a community sample of married couples, as degree of acquaintance increased, self-other agreement about extent of pathology increased. As noted earlier, there is also a research literature on the tendency for individuals to rate themselves higher on maladaptive cognitive and affective variables than do those who know them well. For example, there is a bias toward more favorable (less negative) other-reports than self-reports in the domain of personality pathology (Vazire, 2010).

If self-talk is seen as a maladaptive behavioral characteristic, then we would expect that people will self-report higher levels of self-talk than what they will report for their partners. However, as noted earlier, Brinthaupt et al. (2009) found that scores on the STS were only weakly related to social desirability scores. In addition, research shows that people generally use less private speech and more inner speech as they move from childhood to adulthood (Winsler and Naglieri, 2003; Duncan and Tarulli, 2009). Thus, adult self-talk tends to be hidden from others, which should also create a tendency toward higher self-reported than other-reported self-talk scores.

In Study 3, we examined the extent to which the reports of others correspond with self-reported self-talk frequency. For this study, pairs of participants, who either knew their partner well or did not know their partner at all, rated themselves and the other on the STS as well as a measure of private speech (out loud self-talk). The stranger data served as the control condition, providing a baseline for what respondents think about how often people in general normally talk to themselves. We expected that close others would show greater self-other agreement on self-talk frequency than would strangers. We also expected that, for those who know each other, increased relationship closeness would be associated with increased levels of self-talk frequency agreement. These predictions are based on the assumption that greater relationship closeness will provide partners with more situations where self-talk might occur and more accurate information about how often self-talk occurs, compared to strangers. Finally, because of its greater observability, we expected stronger self-other agreement results for private speech (i.e., out loud self-talk) than for self-talk measured with the STS (i.e., both inner and private speech).

### Method

#### Participants

Eighty-eight students (44 pairs) participated in the study. Participants were drawn from the department’s General Psychology research pool. They received course credit for their participation. The sample includes 29 men and 59 women, with an average age of 19.75 years (SD = 1.80). With respect to ethnicity, 56% were Caucasian and 35% were African–American. There were 26 pairs in the friends group (18 men, 34 women) and 18 pairs (11 men, 25 women) in the stranger group. Eighteen (69%) of the friend pairs were same-sex and nine (50%) of the stranger pairs were same sex. Forty-two (81%) of the friend group participants identified themselves as friends, siblings, or roommates, with nine participants (17%) indicating an exclusive or non-exclusive dating relationship.

#### Measures

Participants completed the STS and a measure of out loud private speech for both themselves and another person, as well as demographic items and partner ratings. The private speech measure was Duncan and Cheyne’s (1999) Self-Verbalization Questionnaire (SVQ). This is a 27-item measure of activities and situations in which people might talk out loud to themselves. The SVQ consists of four factors, including spatial-search (e.g., “I sometimes verbalize my thoughts when I’m searching for a book in a library”), behavioral-organizational (e.g., “I sometimes think out loud to myself when I’m trying to clean up a mess in a big hurry”), cognitive-attentional (e.g., “I sometimes verbalize my thoughts when I’m memorizing something for an exam”), and affective (e.g., “I sometimes verbalize my thoughts when I’m feeling angry or upset about something”). Respondents rate the items using a seven-point scale (1 = *strongly disagree*, 7 = *strongly agree*). Items are summed to create the subscale and total scores, with possible total scores ranging from 27 to 189. We used only the total scores for this study. The authors report acceptable reliability and validity for the SVQ. In the current sample, the alpha coefficients for the overall SVQ were in the acceptable range for both self-ratings (0.92) and other-ratings (0.93). The alpha coefficients for the overall STS were also in the acceptable range for both self-ratings (0.89) and other-ratings (0.88).

To determine the accuracy of respondents’ self-reports, we created an absolute percentage error (ape) score for the STS and SVQ. The apeSTS was calculated as the absolute value of the difference between the partner’s rating of the participant’s STS score and the participant’s self-reported STS score, divided by the participant rating, and then multiplied by 100. The apeSVQ score was calculated as the absolute value of the difference between the partner’s rating of the participant’s SVQ score and the participant’s self-reported SVQ score, divided by the participant rating, and then multiplied by 100. A smaller ape score represented greater similarity in the ratings of the two partners. Similar measures have been used by researchers to assess measurement accuracy in other domains (e.g., Kang et al., 2012).

For the two self-talk measures, we also calculated a measure of bias – the difference between the partner’s rating of the participant’s STS or SVQ score and the participant’s self-reported STS or SVQ score, divided by the participant rating, and multiplied by 100 (biasSTS and biasSVQ, respectively). Bias scores in the positive direction indicated a bias toward reporting higher self-talk scores for the other than for self. Finally, we calculated a simple difference score for each measure – the difference between the partner’s score for the participant and the participant’s STS or SVQ score (diffSTS and diffSVQ, respectively). Difference scores in the positive direction reflected higher other-reported than self-reported self-talk scores.

Demographic items included participant age, gender, and ethnicity. Participants in the significant other condition indicated the nature of the relationship with their friend/partner (e.g., friend, roommate, dating), whether they currently lived with this person, and how long they had known them. Next, they indicated how much time in an average day and an average week they spent in the physical presence of their partner. Finally, participants in both the significant other and stranger conditions rated, using five-point scales, how close they and their partner were (1 = *not close at all*, 5 = *extremely close*), how well they knew their partner (1 = *not very well at all*, 5 = *extremely well*), how well they understood how their partner thinks about him/herself (1 = *not very well at all*, 5 = *extremely well*), how well they would say that their partner knows them (1 = *does not know me very well at all*, 5 = *knows me extremely well*), and how well they would say that their partner understands how they think about themselves (1 = *not very well at all*, 5 = *extremely well*).

#### Procedure

Prior to the testing session, we informed participants in the significant other condition to bring a friend, romantic partner, or close other with them in order to participate and receive course credit. Participants in the stranger condition were randomly assigned a partner at the start of the testing session. We collected data for the two conditions in separate testing sessions, with all participants in each session falling in the same condition. Participants first completed the self-talk/private speech measures for themselves and their partner (in random order), followed by the demographic and other items.

### Results and Discussion

#### Descriptive Statistics

Participants in the friend group differed significantly from participants in the stranger group in the expected direction on all of the relationship measures. For example, compared to the stranger participants, the friend participants reported being closer to their partners [*M*_F_ = 4.12, SD_F_ = 0.96; *M*_S_ = 1.08, SD_S_ = 0.28; *t*(86) = 18.33, *p* = 0.000] and knowing their partners better [*M*_F_ = 3.98, SD_F_ = 1.02; *M*_S_ = 1.00, SD_S_ = 0.00; *t*(86) = 17.52, *p* = 0.000], as well as their partners knowing them better [*M*_F_ = 3.92, SD_F_ = 0.98; *M*_S_ = 1.03, SD_S_ = 0.17; *t*(86) = 17.32, *p* = 0.000].

We examined the correlations among the STS and SVQ ratings for the entire sample. Self-rated STS scores were highly correlated with participants’ ratings of their partners, *r*(86) = 0.688, *p* < 0.001. Similarly, self-rated SVQ scores were highly correlated with participants’ ratings of their partners, *r*(86) = 0.695, *p* < 0.001. These results suggest that participants assumed that their own levels of self-talk were similar to the levels likely to be shown by their partners. In addition, self-rated STS and SVQ scores were significantly correlated, *r*(86) = 0.503, *p* < 0.001, as were other-rated STS and SVQ scores, *r*(86) = 0.608, *p* < 0.001. These results indicate substantial overlap between the two self-talk measures.

The apeSTS scores for the entire sample (*M* = 21.63, SD = 21.74) differed significantly from 0 (perfect accuracy), *t*(87) = 9.33, *p* < 0.001, as did the apeSVQ scores (*M* = 23.86, SD = 19.95), *t*(87) = 11.22, *p* < 0.001. These results indicated that participants tended to rate themselves differently on self-talk compared to how their partners rated them. The bias and difference scores for the entire sample did not differ significantly from 0.

#### Comparison of Friend and Stranger Conditions

**Table 4** presents data for the self-talk measures. As the table indicates, comparison of the two groups on the accuracy measures revealed one significant difference – the friend group showed larger average percentage error scores than the stranger group for the STS. This finding was opposite to what we expected. Because we failed to find any differences on the bias or difference measures, the apeSTS results suggest that participants in the friend condition were generally less accurate in rating their partner’s self-reported self-talk frequency than those in the stranger condition, but not in a specific (over- or under-reporting) direction. Separate paired-sample *t*-tests for each group indicated that the friend group did not differ in their self- and other-ratings on the STS or SVQ. However, the stranger group reported significantly higher self-ratings (*M*_s_ = 58.19, SD_s_ = 8.44) than other-ratings (*M*_o_ = 54.81, SD_o_ = 7.36) for the STS, *t*(35) = 2.66, *p* = 0.012. Strangers also reported significantly higher self-ratings (*M*_s_ = 125.44, SD_s_ = 21.74) than other ratings (*M*_o_ = 117.42, SD_o_ = 21.22) for the SVQ, *t*(35) = 2.66, *p* = 0.012. Thus, there was a tendency toward more frequent self-rated self-talk than other-rated self-talk for the strangers but not for the friends.

**Table 4:** Study 3: Comparison of friend and stranger groups on major measures.

	Friend (*n =*52)		Stranger (*n* = 36)			
	Mean	SD	Mean	SD	*t*	*p*
STS Self	57.54	12.52	58.19	8.44	0.274	0.785
STS Other	56.94	11.87	54.81	7.36	0.959	0.340
SVQ Self	123.19	29.56	125.44	21.74	0.390	0.698
SVQ Other	122.88	28.73	117.42	21.22	0.972	0.334
apeSTS	25.76	26.19	15.65	10.65	2.192	0.031
biasSTS	4.79	36.60	-3.80	18.72	1.296	0.199
diffSTS	-0.60	16.79	-3.31	10.86	0.852	0.397
apeSVQ	24.69	22.58	22.66	15.61	0.468	0.641
biasSVQ	4.83	33.28	-2.91	27.62	1.147	0.254
diffSVQ	-0.31	31.76	-8.03	33.40	1.098	0.275

STS, Self-Talk Scale; SVQ, Self-Verbalization Questionnaire; “ape” denotes average percentage error between self and other; “bias” denotes general direction of disagreement; “diff” refers to difference between total scores. Positive bias and diff scores reflect higher other-reported self-talk.

Additional analyses examining only the friend group indicated that level of closeness was unrelated to the various accuracy scores (all *p*s > 0.28), that participants who lived with their partner did not differ from those who did not live together on those measures (all *p*s > 0.55), and that those in dating relationships did not differ in their accuracy scores from those in non-dating relationships (all *p*s > 0.23). Thus, contrary to our prediction, relationship closeness was unrelated to the degree of self-other self-talk accuracy.

We also analyzed the correlations among the major measures separately for the two groups. For the friend group, self-reported and other-rated scores were highly correlated for both the STS [*r*(50) = 0.740, *p* < 0.001] and the SVQ [*r*(50) = 0.727, *p* < 0.001]. For the stranger group, self-reported and other-rated scores were also significantly correlated for both the STS [*r*(34) = 0.538, *p* = 0.001] and the SVQ [*r*(34) = 0.644, *p* < 0.001]. However, the Fisher *r*-to-*z* transformation showed that the correlations did not differ significantly for either the STS (*z* = 1.55, *p* = 0.12) or the SVQ (*z* = 0.70, *p* = 0.48).

In summary, we found no evidence that people who know others well showed greater accuracy in rating their partner’s typical self-talk levels than did complete strangers. In fact, friends were less accurate in rating their partner’s self-reported self-talk frequency than were strangers. There was also no support for the prediction of greater self-other agreement with the private speech measure compared to the STS. The results suggest that when people rate others who they know on their self-talk levels, the raters may be relying more on their assumptions about their partner’s typical self-talk than on behavioral observations of the phenomenon. The results are similar to the findings of Carlson et al. (2013), who found that self-reported internalizing personality characteristics were more accurate than informant reports of those characteristics.

All participants seemed to have assumed that their partners talk to themselves as frequently or infrequently as the participants themselves do. There was a tendency toward greater similarity in self-talk scores among friends than strangers. Thus, the greater inaccuracy shown by the friends might be attributable to their assumption that their partners were more similar to themselves than what the strangers assumed about their randomly assigned partners.

This study focused on the question of the agreement between self- and other-reports of self-talk frequency. Results were similar for both self-talk measures. With respect to the validity of the STS or SVQ, it appears that talking to oneself either silently or aloud is not a behavior that friends can accurately assess. The results suggest that relying on other-reports of self-talk frequency is not an effective way to determine the validity of self-talk measures. It is likely that instances of observing or learning about a friend’s self-talk episodes are infrequent; future research examining the nature and extent of friends’ knowledge of their partner’s self-talk would provide additional insight into the relationship between self- and other-reports of self-talk frequency.

## General Discussion

The purpose of this set of studies was to determine the accuracy of STS scores. Results from the first two studies provided good support for the argument that self-reports of typical levels of self-talk frequency correspond well with recent situations where participants reported that self-talk had occurred. In particular, Study 1 results showed that respondents’ STS scores were consistent with their reports about specific recent experiences where self-talk occurred. The results from Study 2 showed that frequent self-talkers were more likely to report having talked to themselves when STS-related situations occurred, compared to infrequent self-talkers. Study 3 findings indicated that people who knew each other well were less accurate in their assessments of their partner’s typical self-talk frequency than were strangers. The latter findings indicate that there may be reasons to doubt the accuracy of knowledgeable informants’ assessments of another’s typical self-talk.

Consideration of the STS subscales revealed some interesting patterns and differences. In Study 1, all the STS subscales were significantly correlated with reports of the occurrence of self-talk in recent situations. However, in Study 2, self-reported self-critical and social-assessing self-talk frequency were similar for frequent and infrequent self-talkers. Individuals appear to have less difficulty in accurately judging their typical self-reinforcing self-talk frequency than more negative self-talk. It may be the case that self-reinforcing self-talk is associated with less internal conflict or fewer alternative interpretations compared to self-critical or social-assessing self-talk. It is also the case that some STS items refer explicitly to self-talk about communicating with others (e.g., social-assessing and self-managing items pertaining to what the respondent should do or say). Self-talk that pertains to talking with others may be qualitatively different from self-talk pertaining to purely intrapersonal situations. Research on the phenomenological and social interaction aspects of different kinds of self-talk would be an interesting extension of the current findings.

There is a great deal of research documenting that a lack of insight is characteristic of many mental disorders (Oltmanns and Powers, 2012). The distinction between *ego-dystonic* (personal characteristics that conflict with one’s self-image) and *ego-syntonic* (characteristics that are consistent with one’s self-image) is also relevant to the question of how people view their self-talk. As Oltmanns and Powers (2012) noted, many mental disorders are ego-dystonic, whereas most personality disorders are ego-syntonic. If people consider talking to themselves as an indication of a mental disorder, they may under-report its frequency. If, however, they view self-talk as a personality characteristic (as seems to be the case based on the present findings), they should be less inclined to under-report its frequency. In the present set of studies, we did not examine participants’ beliefs about or perceptions of the phenomenon of self-talk. The results from Study 3 suggest that self-talk is viewed by people as ego-syntonic. Addressing how people view the phenomenon of self-talk (e.g., their stereotypes or assumptions about it) and how those perceptions are related to their self-reported frequency is a promising area for future research.

Another direction for future research would be to manipulate the description of the phenomenon of self-talk when it is being measured. For instance, the instructions for the STS state that “[r]esearchers have determined that all people talk to themselves, at least in some situations or under certain circumstances.” The perceived appropriateness of talking to oneself may affect the self-reported nature and frequency of self-talk. We would expect that, if informing respondents that researchers have determined that “mentally disturbed people talk to themselves,” significant under-reporting of self-talk frequency would likely occur.

The question of why people show individual differences in self-reports of self-talk frequency is an intriguing one. Early childhood experiences might contribute to such differences (e.g., Brinthaupt and Dove, 2012). Self-reports of self-talk might also reflect respondents’ awareness of the situations where self-talk occurs. Individual differences in the motivation to recognize or acknowledge one’s self-talk might account for some of the differences in STS scores (see Chin et al., 2012). Future research could help to determine the extent to which self-reports of self-talk reflect actual frequency differences rather than differences due to beliefs or motivations with respect to this inner experience.

In their critique of ESM and questionnaire approaches to studying inner experience, Hurlburt and Heavey (2015, p. 156) note that how people answer the prompts or rate the scale items is likely to be a generalization based on “an unspecified mixture of heuristic (recency, availability, etc.), supposition, confirmation bias, and so on.” A more accurate understanding of the STS and the present results would be to note that the STS is a measure of whether people notice talking to themselves and how often they recall doing so upon reflection. The present results refer more to respondents’ interpretations of “experience and generalities” (Hurlburt and Heavey, 2015, p. 156) than actual, ongoing experiences of talking to themselves. Of course, the methods used in the present studies were designed to assess the validity of the STS rather than the nature and content of currently ongoing self-talk. Based on the results, researchers who use the STS can be confident that its scores are related to respondents’ self-reported instances of talking to themselves across different situations. The extent to which the scores on the STS correspond to actual instances of “pristine” inner experiences remains to be seen.

Taken together, the current findings indicate that general self-reported STS scores are good approximations of specific reports of self-talk. Combined with other research supporting the psychometric properties of the STS, the research reported here provides evidence that this measure of self-talk frequency can be used successfully to study individual differences in the phenomenon of self-talk.

## Conflict of Interest Statement

The authors declare that the research was conducted in the absence of any commercial or financial relationships that could be construed as a potential conflict of interest.
